# From Deficits in Emotional Intelligence to Eating Disorder Symptoms: A Sequential Path Analysis Approach Through Self-Esteem and Anxiety

**DOI:** 10.3389/fpsyg.2021.713070

**Published:** 2021-08-18

**Authors:** María Angeles Peláez-Fernández, Juana Romero-Mesa, Natalio Extremera

**Affiliations:** Department of Social Psychology, Social Work, Social Anthropology, and East Asian Studies, Faculty of Psychology, University of Málaga, Málaga, Spain

**Keywords:** emotional intelligence, eating disorders, anxiety, self-esteem, path analysis

## Abstract

Past studies have reported emotional intelligence (EI) as a relevant factor in development and maintenance of eating disorders (ED), as well as in increasing self-esteem and reducing anxiety. Similarly, research has showed that anxiety and self-esteem are positively and negatively associated to ED criteria, respectively. However, no prior studies have yet tested the multiple intervening roles of both self-esteem and anxiety as potential mediators of the association between EI and ED symptomatology. The present study aims to bridge these gaps by testing a sequential path model. Specifically, we examine the potential sequential mediation effects of self-esteem-anxiety on the link between EI and ED. A sample composed of 516 Spanish undergraduate students and community adults completed measures of EI, self-esteem, anxiety, and ED symptomatology. The results show that high levels of EI were positively associated with self-esteem and negatively associated with anxiety and ED symptoms. Anxiety was positively associated to ED symptoms, while self-esteem levels were negatively linked to ED symptoms. Moreover, path analyses showed that self-esteem and anxiety fully mediated the relationship between EI and ED symptoms in sequence. These findings suggest that EI plays a key role in reducing symptomatology of ED through increased self-esteem and reduced anxiety symptoms, providing novel evidence regarding psychological mechanisms through which EI contributes to a reduction of ED symptomatology. Implications for assessing and improving these psychological resources in ED preventive programs are discussed.

## Introduction

Eating disorders (ED) are persistent disturbances of eating or eating-related behaviors that result in significant impairments in psychosocial functioning and physical health (American Psychiatric Association (APA), 2013). In the last two decades, the worldwide prevalence of ED has increased from 3.5 to 7.8% (Galmiche et al., 2019), and the rate remains significantly higher among females (American Psychiatric Association (APA), 2013). Anxiety disorders and depression are among the most common comorbid diagnoses in ED (Godart et al., 2002, 2007).

Theoretical background and empirical evidence have supported the idea that deficits in the processing and managing of emotions play a key role in the development and maintenance of ED (Polivy and Herman, 1993) and that difficulties with the regulation of emotions are related with ED psychopathology (Corstorphine, 2006; Fox, 2009; Haynos and Fruzzetti, 2011; Lavender et al., 2015; Rowsell et al., 2016). For example, some meta-analytic findings have confirmed that high levels of negative emotionality increase the risk of eating pathology (Stice, 2002). Accordingly, consistent with the transdiagnostic cognitive-behavioral model for ED (Fairburn et al., 2003), mood changes (along with external events) play a relevant role in both the maintenance and relapse of ED. In fact, a broad form of enhanced cognitive-behavioral therapy (CBT-Eb; Fairburn et al., 2009), including mood intolerance, clinical perfectionism, low self-esteem, and interpersonal difficulties, along with the well-established cognitive-behavioral therapy (CBT) for ED (National Institute for Clinical Excellence (NICE), 2004; Wilson, 2005) have shown better results compared to CBT alone, both for ED patients with minor and subclinical symptoms and for those with more relevant psychopathology symptoms (CBT-Eb; Fairburn et al., 2009). Similarly, the cognitive-emotional-behavioral therapy (CEBT) (Corstorphine, 2006), including assessment of emotions and emotional management techniques, has been shown to improve participants' emotional eating behaviors, as well as their self-esteem, depression, and anxiety (Campbell, 2012).

### Emotional Intelligence and Eating Disorders

Emotional intelligence (EI) is a relatively new construct related to emotions that comprises a set of basic emotional skills. From an ability perspective, EI is defined as “the ability of people to perceive, use, understand and manage emotions” (Mayer and Salovey, 1997; p. 532). This EI theoretical framework involves four basic emotional dimensions: emotional perception and expression, emotional facilitation, emotional understanding, and emotional regulation. From this perspective, emotional regulation is defined as the ability to manage both positive and negative emotions in themselves and in others, integrating emotion and cognition effectively (Mayer et al., 2016). These emotional skills can be developed in clinical settings through systematic and comprehensive EI training (Mayer et al., 2000; Kotsou et al., 2018).

In the last decade, an emerging line of research has typically shown the significant relationship between EI and ED symptoms. For example, some prior research has found that individuals with some EI deficits are more prone to display disordered eating attitudes and behaviors (Costarelli et al., 2009; Pettit et al., 2009; Filaire et al., 2010; Zysberg and Rubanov, 2010; Hambrook et al., 2012; Zavala and López, 2012; Zysberg, 2013; Zysberg and Tell, 2013; Gardner et al., 2014; Koch and Pollatos, 2015; Cuesta et al., 2017; Peres et al., 2018; Foye et al., 2019). In addition, recent systematic reviews have reported that these abilities are relevant factors in both the development and maintenance of ED (Romero-Mesa et al., 2020; Giusti et al., 2021). These findings provide some preliminary support for the role of emotions in disordered eating attitudes with a view to the prevention and management of ED and point to the potential use of EI measures to identify individuals at risk of ED.

### Self-Esteem and Anxiety as Mediators

Beyond this direct association between EI and ED symptoms, other potential underlying processes have been theorized through which EI might impact ED symptoms. Two of these individual psychological mechanisms considered to be relevant mediators might be self-esteem and anxiety. A growing body of research has supported the theory that deficits in emotional skills are significant predictors of reduced self-esteem domains (Fernández-Berrocal et al., 2006; Hasanvand and Khaledian, 2012; Bibi et al., 2016). Likewise, it has been found that people with high levels of anxiety typically report difficulty in accurately perceiving, using, understanding, and managing their own emotions (Fernández-Berrocal et al., 2006; Connor and Slear, 2009; Kousha et al., 2018). Taken together, these findings suggest that people with high levels of EI feel more security and less stress and also believe in their abilities, showing higher feelings of self-worth, goodness, and self-respect.

On the other hand, there is some theoretical and empirical support that self-esteem and anxiety are risk factors for ED symptoms. As mentioned above, improving self-esteem in combination with other components resulted in a more effective treatment for ED patients compared to CBT therapy alone. In addition, several studies have consistently corroborated a negative relationship between self-esteem and ED criteria (Silverstone, 1990, 1992; Silvera et al., 1998). Besides, numerous studies have found that high levels of anxiety are a well-recognized symptom in individuals with ED (Arnow et al., 1995; Levinson and Rodebaugh, 2012; Menatti et al., 2015) and that anxiety worsens the ED psychopathology (Brand-Gothelf et al., 2014). Given the high comorbidity between ED and anxiety disorders and the fact that both disorders share the same components (evaluative, affective, and somatic; Kaye et al., 2004), the robust relationship between ED and anxiety is not surprising.

Along with the individual role of self-esteem and anxiety in ED symptomatology, several studies have tested the combined role of self-esteem and anxiety on ED. For example, a recent study found that self-esteem (together with mood dysregulation) moderated the association between levels of anxiety/depression and greater deterioration of ED (Sander et al., 2021). In addition, Aloi and Segura-García (2016) found that low self-esteem had an indirect effect on the risk of developing an ED through the mediating action of anxiety.

There is also evidence that anxiety and self-esteem (considered independently) play a relevant mediating role between EI and ED symptoms. For example, prior findings have found that social anxiety mediated the relationship between EI and ED risk (Li, 2018). Thus, Hambrook et al. (2012) found that self-reported anxiety levels mediated the observed relationship between EI and anorexia nervosa (AN).

### Purpose of the Present Research

In sum, there is empirical support for the link between EI, higher self-esteem, and reduced anxiety, as well as between levels of self-esteem and anxiety and ED symptomatology. It has also been pointed out that individuals with higher EI use their ability to maintain a global feeling of self-worth when appropriate and effectively manage a distressed mood when faced with negative events that are considered key in the development of ED symptoms. Hence, the assessment of self-esteem and anxiety as potential mediators in the association between EI and the symptomatology of ED seems to be justified (Hambrook et al., 2012; Li, 2018). However, most of these studies only examined the role of a single mediator in the linkage EI-ED symptomatology and have rarely evaluated effects of the mediators concurrently. Specifically, and to the best of our knowledge, no prior research has examined the cumulative effect of self-esteem and anxiety on the relationship between EI and ED symptomatology. Including these mediators in the serial model would help unveil the underlying mechanisms through which EI influences ED and would also help clinicians and educators to focus on the factors with the most clinical relevance for the prevention and treatment of ED symptomatology. Therefore, the purpose of the present study is 2-fold: first, we sought to examine the relations between EI, self-esteem, anxiety, and ED symptomatology. Second, we sought to determine whether self-esteem and anxiety mediated the relation between EI and ED symptoms in sequence. Since prior studies have found that individuals with higher self-esteem are more likely to have lower levels of anxiety (Sowislo and Orth, 2013), and that self-esteem has an indirect effect on ED risk, as mediated by anxiety (Aloi and Segura-García, 2016), we expected that both mediators might act in this sequence; that is, individuals with high positive cognitions about themselves would have lower levels of anxiety, which in turn would lead them to an amelioration of ED symptomatology. Overall, considering prior research on the significant associations between EI, self-esteem, anxiety and ED symptoms, we developed the following research hypotheses:

**Hypothesis 1**. EI is positively associated with higher self-esteem and negatively linked to anxiety and ED symptoms.
**Hypothesis 2**. (Single mediation). EI predicts higher levels of self-esteem and lower levels of anxiety. These variables, in turn, independently predict lower levels of ED symptomatology.
**Hypothesis 3**. (Sequential mediation). Self-esteem and anxiety might serve as mediators in a sequential mediation model between EI and ED symptoms; that is, EI positively predicts self-esteem, leading to lower levels of anxiety, further decreasing ED symptomatology.

## Materials and Methods

### Participants

The study sample consisted of 516 Spanish undergraduate students and community adults (319 females and 197 males) located in Southern Spain. Their ages ranged from 18 to 77 years, with a mean age of 38.89 years (*SD* = 14.76) (see Table 1). Given that the prevalence of ED in elderly women (aged 65–94 = 3.25%) has been found to be comparable to young women (Conceição et al., 2017) and that, according to a systematic review, late-life onset ED do continue to occur in the elderly (Lapid et al., 2010), we have not excluded older people from our study.

**Table 1:** Sample distribution by age and sex.

**Age intervals**	**Men**	**Female**	**Total**
18–29	48	129	177
30–39	34	43	77
40–49	35	55	90
50–59	62	79	141
60–69	17	11	28
70 and older	1	2	3
Total	197	319	516

The educational level in the present sample was as follows: 15 (2.9%) had no formal education, 73 (14.1%) had a primary-level education, 120 (23.3%) had not completed secondary school, 57 (11.0%) had completed secondary school, 186 (36.0%) had completed University Studies, and 65 (12.6%) had post-graduate studies.

The percentage of at-risk participants (i.e., those scoring ≥ 20 in the EAT-26) was 6.2% (32 out of 516).

### Procedure

University and community participants were solicited using non-probabilistic convenience sampling techniques via an online survey format. A student-recruited sampling methodology was used following guidelines by Wheeler et al. (2014), which allowed us to access a community sample from a University setting. The community participants were recruited with the assistance of students enrolled in a psychology course at university, who were asked to recruit at minimum two adults over the age of 30 through their personal network and then administer the online version of the questionnaires to them. The online survey was designed so that incomplete questionnaires could not be saved, which allowed only the whole completed questionnaires to be received. Student participants earned points for their participation in the study. Before getting to the online survey, participants were informed that the survey was about eating habits and emotions, and that their participation was entirely voluntary. All participants provided written informed consent according to the Declaration of Helsinki. The procedure was approved by the ethics committee of the University of Málaga (104-2020-H). The administration procedure lasted for ~30 min. The sample was obtained from May 2019 to November 2020. The percentage of participation among students was of 78.28%.

### Instruments

The following well-validated measures were used.

#### Wong Law Emotional Intelligence Scale

The Wong Law Emotional Intelligence Scale (WLEIS; Wong and Law, 2002), in its Spanish version (WLEIS-S; Extremera et al., 2019), was selected as a self-reported ability EI scale based on the theoretical framework of Mayer and Salovey (1997). The WLEIS is a short and cost-free EI measure that consists of 16 items that measure four EI aspects: appraisal of one's own emotions, appraisal of others' emotions, use of emotion, and regulation of emotion, e.g., “I always tell myself I am a competent person.” Questions are scored on a 7-point scale ranging from totally disagree to totally agree. Total scores range from 16 to 112. Higher scores indicate a higher level of EI (Extremera et al., 2019). In this study, Cronbach's alpha was 0.91.

#### Eating Attitudes Test

The Spanish version of the Eating Attitudes Test (EAT-26; Garner et al., 1982; Rivas et al., 2010) was used as a measure of risk of ED and the presence of disordered eating attitudes. The EAT-26 is a non-clinical self-report 26-item measure; e.g., “feel extremely guilty after eating.” It includes three subscales that measure: (1) diet and concern for thinness, (2) bulimia and concern for food, and (3) oral control. Questions are scored on a 6-point scale ranging from “always” scoring 3, “most often” scoring 2, “often” scoring 1, “sometimes” scoring 0, “rarely” scoring 0, and “never” scores as 0. Only one of its items scores reversely. The range of the total score is 0–78. A total score ≥ 20 often indicates a risk of ED. A higher score indicates a higher risk of ED. The Spanish version has shown satisfactory reliability (Rivas et al., 2010). Cronbach's alpha was 0.80.

#### Rosenberg Self-Esteem Scale

The Rosenberg Self-Esteem Scale (RSE; Rosenberg, 1965), in its Spanish version (RSES; Martín-Albo et al., 2007), was selected as a self-reported scale for direct evaluation of global self-esteem. The RSE contains brief statements that reflect general feelings about oneself. It consists of 10 items that are answered on a 4-point Likert scale (from totally agree to totally disagree); e.g., “I wish I could have more respect for myself.” For correction, the scores of five of its items must be inverted. The total score ranges from 10 to 40. Scores ≥ 30 indicate optimal self-esteem, between 26 and 29 indicate average self-esteem (needs improvement), and scores ≤ 25 points indicate significant self-esteem problems (Martín-Albo et al., 2007). Cronbach's alpha was 0.78.

#### Depression Anxiety Stress Scales

The self-reported anxiety dimension of the Spanish version of the Depression Anxiety Stress Scale (DASS-21; Lovibond and Lovibond, 1995; Bados et al., 2005) was used. It consists of 7 items with a Likert-type response format with four alternatives, which are ordered on a scale from 0 to 3 points; e.g., “I felt scared without any good reason.” The score varies between 0 and 21 points. The recommended cut-off scores for severity labels are: 0–7 = normal; 8–9 = mild; 10–14 = moderate; 15–19 = severe; 20+ = extremely severe. Cronbach's alpha in this study was 0.82.

### Analytical Strategy

After calculating descriptive statistics and computing the bivariate correlation between EI, self-esteem, anxiety, and ED symptoms, the SPSS macro PROCESS (Hayes, 2018) was used to conduct multiple mediation analyses for testing the potential mediating role of self-esteem and anxiety in the linkage between EI-ED symptomatology. A bootstrapping method with 5,000 esteem resamples was used to calculate overall indirect effects and specific indirect effects. The direct and indirect effects are considered to be statistically significant if 95% of the bootstrap confidence intervals do not contain zero. Thus, this procedure enables multiple mediators to be examined and determines the independent effect of each mediator while controlling for the other. Preliminary ANOVA and *t-*tests showed differences according to age and sex, respectively, in ED symptoms, self-esteem and anxiety. Female participants scored significantly higher than males in ED symptoms and anxiety, and lower in self-esteem (all *p*s < 0.01); younger participants scored marginally significantly higher than older ones in ED symptoms and anxiety (*p*s < 0.10) and significantly lower in self-esteem (*p* <0.001). Therefore, sex and age were entered as covariates to control any potential confounding effects.

## Results

### Descriptive Data and Correlations

Descriptive statistics, response ranges, normative data, reliability coefficients, and correlations among the study variables are presented in Table 2. The column “Response ranges” indicates the minimum and maximum possible scores in the questionnaires. The column “Normative data M (SD)” indicates means and standard deviations from normative data. For EI, self-esteem, and anxiety, normative data correspond to the information provided by Spanish adaptations of the questionnaires. For the EAT-26, since the Spanish adaptation of EAT-26 was performed with adolescents, we have included the data from Johnson and Bedford (2004), who employed a Canadian sample comparable to ours (men and women, age range 18–94). As Table 2 shows, EI was positively correlated to self-esteem and negatively associated to anxiety. As expected, self-esteem was significantly and negatively associated with ED symptoms, while anxiety levels showed significant and positive associations with symptoms of ED. Finally, EI was negative and significantly linked to ED symptomatology.

**Table 2:** Descriptive statistics, response ranges, normative data, reliability, and bivariate correlations.

	**M (*SD)***	**Response ranges**	**Normative data M (SD)**	**α**	**1**	**2**	**3**
1. Emotional intelligence	5.15 (0.92)	1–7	5.02 (0.96)	0.91			
2. Anxiety	3.45 (3.66)	0–3	6.02 (5.61)	0.82	−0.33^**^		
3. Self-esteem	29.29 (4.62)	1–4	31.83 (4.23)	0.78	0.51^**^	−0.34^**^	
4. Symptoms of eating disorders	6.24 (7.11)	0–3	7.40 (6.92)	0.80	−0.18^**^	0.24^**^	−0.27^**^

** *p <0.01*.

*M, Mean; SD, Standard Deviation; α, Cronbach's alpha*.

### Serial Mediational Analysis

We examined whether the relationship between EI and ED symptoms was sequentially mediated by self-esteem and anxiety. Both age and sex were added as control variables. Results of the mediation analyses are presented in Table 3.

**Table 3:** Testing the pathways of the serial mediation model.

		**95% bias-corrected CI**	
**Mediation analysis path**	***b***	**Lower**	**Upper**
Total effect	−0.16^a^	−1.905	−0.604
Direct effect	−0.03	−0.957	0.505
Total indirect effect	−0.14^a^	−0.195	−0.081
EI → Self-esteem → ED symptoms	−0.09^a^	−0.145	−0.035
EI → Anxiety → ED symptoms	−0.03^a^	−0.066	−0.009
EI → Self-esteem → Anxiety → ED symptoms	−0.02^a^	−0.031	−0.005
Model *F*_(5, 510)_ = 12.09; *p* <0.001; *R* = 0.33; *R*^2^ adj = 0.11			

*All paths were estimated while controlling for age and sex; Standardized regression coefficients shown for each path*.

*EI, Emotional Intelligence; ED, Eating Disorders*.

a *Empirical 95% confidence interval does not include zero*.

**Figure 1:**
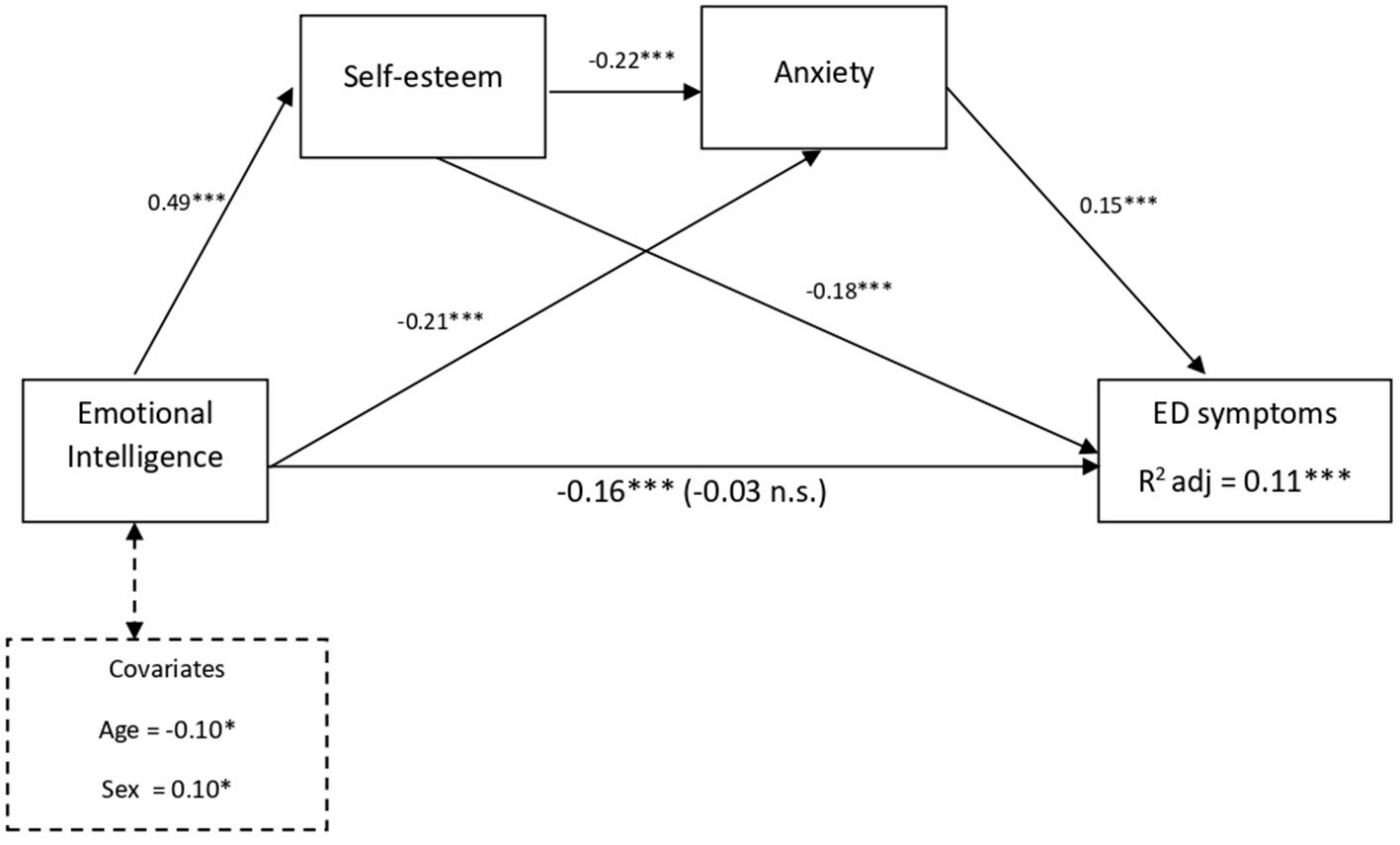
Multiple mediation model for the effect of EI and ED symptoms *via* self-esteem and anxiety controlling for age and sex as covariates. Total effect (c-path) is given in parentheses; standardized coefficients; *p < 0.05; ***p < 0.001.

As shown in Figure 1, the three hypothetical mediating effects were supported. First, the specific indirect effects of EI on ED symptoms through self-esteem [EI → Self-esteem → ED symptoms] were supported (B = −0.09, *SE* = 0.03; 95% CI = −0.15, −0.04). Second, anxiety was found to mediate the association between EI and ED symptoms [EI → Anxiety → ED symptoms] (B = −0.03, *SE* = 0.01; 95% CI = −0.066, −0.009). Third, the sequential pathway of EI → Self-esteem → Anxiety → ED symptoms, was significant (B = −0.02, *SE* = 0.01; 95% CI = −0.031, −0.005). Accordingly, higher levels of EI were serially associated with higher self-esteem, lower anxiety, and finally lower ED symptoms. Thus, the residual direct pathway between EI and ED symptoms was no longer significant (b = −0.03, *p* = 0.578). Therefore, self-esteem and anxiety fully mediated the link between EI and ED symptoms. This final serial mediation model was significant, accounting for 11% of the variance in ED symptoms [*R*^2^ adj = 0.11; *F*_(5, 510)_ = 12.08; *p* <0.001].

## Discussion

The present study examined whether self-esteem and anxiety, which previous literature confirm as relevant variables related to ED symptomatology, mediated the relationship between EI and ED symptomatology within a Spanish college student and community sample.

The results of path analyses showed that self-esteem and anxiety play a fully sequential mediating role between EI and ED symptomatology, suggesting that EI was positively linked to higher self-esteem and lower anxiety, which in turn predicted lower levels of ED symptoms. The findings support the notion that EI decreases ED symptoms indirectly, suggesting that higher self-esteem and decreased anxiety may be possible underlying mechanisms through which emotional abilities contribute to reducing ED symptoms. These results are consistent with previous studies that found a mediating role for anxiety in the relationship between EI and ED (Hambrook et al., 2012; Li, 2018). Moreover, the serial mediation was also significant, suggesting that EI is associated with greater self-esteem, which subsequently reduces anxiety, thus predicting lower ED symptoms. These findings agree with previous meta-analytic research corroborating the robust effect of negative feelings of self-worth on anxiety (Sowislo and Orth, 2013). Our results are also consistent with past studies showing that anxiety mediates the role of self-esteem on the development of ED symptoms (Aloi and Segura-García, 2016) and contributes to the current literature by extending our understanding of the mechanism that underlies the linkage of EI and ED symptomatology.

The present study extends the understanding of the role of EI, self-esteem, and anxiety on ED symptoms in several ways. It provides further support for researchers who argue that low levels of self-esteem (Silverstone, 1990, 1992; Silvera et al., 1998) and high levels of anxiety (Arnow et al., 1995; Kaye et al., 2004; Levinson and Rodebaugh, 2012; Menatti et al., 2015) are significantly associated with ED symptoms. Our findings reinforce these models by suggesting that deficits in EI might lead to reduced levels of self-esteem and high levels of anxiety, which are associated with higher ED symptoms. Our data also support the transdiagnostic CBT model for ED (Fairburn et al., 2003), which includes mood changes as independent variables that affect ED symptomatology, as well as the suitability of including treatment for emotional abilities and low self-esteem, as did the broad form of enhanced CBT (CBT-Eb; Fairburn et al., 2009). Our results also support the suitability of CEBT (Corstorphine, 2006) for the treatment of ED, as this extended version of CBT incorporates the assessment as well as emotion-management techniques. Accordingly, the findings of this study have theoretical implications, as they suggest that alterations in self-esteem might be partially responsible for the mood changes that trigger dysfunctional eating behaviors (transdiagnostic CBT model; Fairburn et al., 2003). These findings also have practical implications for the prevention and treatment of ED. The inclusion of programs on EI training, self-esteem promotion, and anxiety management in school curricula could minimize the acquisition and maintenance of ED in the child-adolescent population. Likewise, clinicians could incorporate EI training and psychoeducation on how emotions relate to ED symptoms, which could help patients increase their ability to understand and manage their emotional states. Therapists could also include specific strategies for anxiety management along with the other components of CBT-Eb (Fairburn et al., 2009) and CEBT (Corstorphine, 2006). All of this could lead to promising and possibly more effective treatments for both ED patients and those at risk of ED. Even less complex ED interventions focused only on increasing emotional competences would enhance individuals' self-esteem, thereby reducing anxiety symptoms, which in turn would lead to an amelioration of ED symptomatology. Moreover, if interventions focused on EI training were reinforced with specific interventions that worked to enhance self-esteem and managing anxiety, the effects of EI on ED symptomatology could be optimized.

The findings of this research should be interpreted in the context of its limitations. Our study used a cross-sectional design, which precludes any causal inference. The order of the variables included in the serial model is based on empirical evidence (Sowislo and Orth, 2013; Aloi and Segura-García, 2016); however, the cross-sectional design of our study precludes causal assumptions. Further research in this area should incorporate longitudinal designs that allow for the study of causal directions between study variables. Also, we used non-clinical and non-representative convenience samples, so it is not possible to generalize these results to clinical samples with diagnoses of ED or to more stratified random samples. Comparing these results with a clinical sample and using cross-validation and random sampling would help confirm whether there are essential differences in the deficits and dynamics of personal resources associated with ED symptoms. Therefore, future studies should incorporate both clinical and non-clinical samples.

Despite its limitations, this research revealed that adults with higher levels of EI (compared to those with low EI) are more likely to have higher levels of self-esteem, which would lead to lower levels of anxiety, which in turn would lead to lower ED symptomatology. These findings open the door to future research concerning the role of emotional competences in ED symptoms; it might be fundamental to consider both levels of self-esteem and anxiety when working with adults who display both deficits in EI and ED symptoms. Also, this research supports the relevance of including training to improve EI skills, foster self-esteem, and reduce anxiety symptoms as a specific treatment for ED or even as additional components of the CBT-Eb.

## Data Availability Statement

The raw data supporting the conclusions of this article will be made available by the authors, without undue reservation.

## Ethics Statement

The studies involving human participants were reviewed and approved by The Research Ethics Committee of the University of Málaga (104-2020-H). The patients/participants provided their written informed consent to participate in this study.

## Author Contributions

MP-F, JR-M, and NE created and organized the study. MP-F and JR-M collected the data. MP-F and NE analyzed the data, critically reviewed the manuscript, and provided constructive comments. JR-M wrote the first draft. MP-F wrote the reviewed draft. All authors contributed to the article and approved the submitted version.

## Conflict of Interest

The authors declare that the research was conducted in the absence of any commercial or financial relationships that could be construed as a potential conflict of interest.

## Publisher's Note

All claims expressed in this article are solely those of the authors and do not necessarily represent those of their affiliated organizations, or those of the publisher, the editors and the reviewers. Any product that may be evaluated in this article, or claim that may be made by its manufacturer, is not guaranteed or endorsed by the publisher.
