# Corrigendum: From Deficits in Emotional Intelligence to Eating Disorder Symptoms: A Sequential Path Analysis Approach Through Self-Esteem and Anxiety

**DOI:** 10.3389/fpsyg.2022.873073

**Published:** 2022-03-17

**Authors:** María Angeles Peláez-Fernández, Juana Romero-Mesa, Natalio Extremera

**Affiliations:** Department of Social Psychology, Social Work, Social Anthropology, and East Asian Studies, Faculty of Psychology, University of Málaga, Málaga, Spain

**Keywords:** emotional intelligence, eating disorders, anxiety, self-esteem, path analysis

In the original article, there was a mistake in Hypothesis 3 and in the Discussion.

In Introduction, Purpose of the Present Research, Hypothesis 3 (Sequential mediation), the word “depression” should be replaced by “ED symptomatology”. The corrected paragraph is shown below.

Hypothesis 3. (Sequential mediation). Self-esteem and anxiety might serve as mediators in a sequential mediation model between EI and ED symptoms; that is, EI positively predicts self-esteem, leading to lower levels of anxiety, further decreasing ED symptomatology.

Similarly, in Discussion, Paragraph 2, the word “depression” should also be replaced by “ED symptomatology”. The corrected paragraph is shown below.

The results of path analyses showed that self-esteem and anxiety play a fully sequential mediating role between EI and ED symptomatology, suggesting that EI was positively linked to higher self-esteem and lower anxiety, which in turn predicted lower levels of ED symptoms. The findings support the notion that EI decreases ED symptoms indirectly, suggesting that higher self-esteem and decreased anxiety may be possible underlying mechanisms through which emotional abilities contribute to reducing ED symptoms. These results are consistent with previous studies that found a mediating role for anxiety in the relationship between EI and ED (Hambrook et al., 2012; Li, 2018). Moreover, the serial mediation was also significant, suggesting that EI is associated with greater self-esteem, which subsequently reduces anxiety, thus predicting lower ED symptoms. These findings agree with previous meta-analytic research corroborating the robust effect of negative feelings of self-worth on anxiety (Sowislo and Orth, 2013). Our results are also consistent with past studies showing that anxiety mediates the role of self-esteem on the development of ED symptoms (Aloi and Segura-García, 2016) and contributes to the current literature by extending our understanding of the mechanism that underlies the linkage of EI and ED symptomatology.

The authors apologize for these errors and state that they do not change the scientific conclusions of the article in any way. The original article has been updated.

## Publisher's Note

All claims expressed in this article are solely those of the authors and do not necessarily represent those of their affiliated organizations, or those of the publisher, the editors and the reviewers. Any product that may be evaluated in this article, or claim that may be made by its manufacturer, is not guaranteed or endorsed by the publisher.

## References

[B1] Aloi M. Segura-García C. (2016). Anxiety and depression mediate the role of low self-esteem and low self-directedness in the development of eating disorders. Int. J. Adolesc. Med. Health. 31:84. 10.1515/ijamh-2016-0084 27740926

[B2] Hambrook D. Brown G. Tchanturia K. (2012). Emotional intelligence in anorexia nervosa: is anxiety a missing piece of the puzzle? Psych. Res. 200, 12–19. 10.1016/j.psychres.2012.05.017 22703722

[B3] Li Y. (2018). Social anxiety and eating disorder risk among Chinese adolescents: the role of emotional intelligence. Sch. Ment. Health. 10, 264–274. 10.1007/s12310-018-9257-4

[B4] Sowislo J. F. Orth U. (2013). Does low self-esteem predict depression and anxiety? A meta-analysis of longitudinal studies. Psychol. Bull 139:213. 10.1037/a0028931 22730921

